# Prevalence of mandibular asymmetries in the pediatric population of Jazan: A radiographic analytical study^☆^

**DOI:** 10.1016/j.heliyon.2024.e32362

**Published:** 2024-06-04

**Authors:** Mohammed Mousa H. Bakri, Satish Vishvnathaiah, Haifa Fathuldeen Bakmani, Abdullah Jaber Hakami, Meshal Saleh Zaidan, Mohammed Abdullah Dighriri, Yaser Ali Jad, Thamer Mohammad Hakami, Hamed Mousa H. Bakri

**Affiliations:** a Division of Oral and Maxillofacial Surgery, Department of Oral and Maxillofacial Surgery and Diagnostic Sciences, College of Dentistry, Jazan University, Jazan City, Saudi Arabia; b Department of Pediatric Dentistry, College of Dentistry, Jazan University, Jazan, Saudi Arabia; c Jazan University, Saudi Arabia; d King Saud University, Riyadh, Saudi Arabia; e College of Dentistry, Jazan University, Jazan, Saudi Arabia; f Department of Periodontology, and Implant Dentistry, Jazan Specialized Dental Center, Jazan City, Saudi Arabia

**Keywords:** Mandibular asymmetry, Pediatric population, Prevalence, Orthopantomogram, Linear dimensions

## Abstract

**Background:**

Facial asymmetry results from variation in mandibular linear and angular dimensions on the right and left sides of the face. Mandibular asymmetry is of great significance to oral surgeons and orthodontists as it directly impacts the facial profile of an individual.

**Aim:**

The present study aimed to measure the prevalence of mandibular asymmetry and its fluctuations during the mixed dentition growth phase in healthy children aged 6–8 years in the Jazan region of Saudi Arabia.

**Method:**

This retrospective observational study was conducted by measuring linear asymmetrical measurements of mandible on orthopantomograms of 390 healthy children (182 boys and 208 girls, aged 6–8 years) with mixed dentition. Linear measurements from orthopantomograms were obtained using a standardized digitizer. Two sets of mandibular measurements were recorded, alongside subjective assessments of mandibular first molar development. An independent *t*-test was employed to assess the significance between measurements on both sides, while one-way ANOVA was used to demonstrate facial asymmetry significance among different age groups.

**Result:**

The result of this study revealed a significant statistical difference (p-value≤ 0.05) for both sides of the mandible across two dimensions: condylar and ramus height (p value = 0.03) and mandibular length (p value = 0.04). The asymmetry index resulted in no asymmetry among most of the included subjects. However, compared to the other three linear measurements, many seven-year-old participants possess mandibular asymmetry on condylar height (54.5 %).

**Conclusion:**

Within the limitation it could be concluded that children in growing age have a significant mandibular asymmetry (mainly 7 years), which, however, is only seldom clinically significant. Hence, treatment plan should be cautiously planned.

## 1 Introduction

Facial asymmetry is a characteristic that can be observed to varying degrees among individuals in the general population. Various factors are associated with this condition: genetics, developing disorder, environmental, neurological, and trauma [1,2]. Mandibular asymmetry is the most common facial asymmetry associated with the growth of an individual and can be identified through methods like clinical evaluation, 2D and 3D imaging, or computed tomography [3].

Asymmetries in the mandible can result from factors such as the adaptive response of the mandible during growth and its deviation during function [4,5]. This adaptation results in change in the shape and size of the glenoid, condyle fossa, and mandibular bone, ultimately causing mandibular asymmetry [1,5]. Studies on cephalometric analysis of growing mandible documented that certain asymmetries are present in children aged 6–12 years, which are widely accepted [[6], [7], [8]]. Moreover, clinical studies investigating craniofacial structure among healthy children have reported asymmetrical patterns on both sides of the mandibular region [5]. Several methods have been proposed to measure mandibular asymmetry, which involves evaluating the vertical and horizontal proportion of the face. These techniques are facial images, radiographs, intraoral scanner, and clinical evaluation [5,6]. Studies have reported that panoramic radiographs are reliable in measuring asymmetries on the mandible's left and right sides [[9], [10], [11]]. Orthopantomograms (OPG) are widely used to analyze pathological conditions of the mandible affecting the condylar process (rheumatoid arthritis) [4,12]. However, these radiographs should be used cautiously while making absolute and relative comparisons as the chances of measurement error are present [4].

Dimensional mandibular asymmetries are mainly associated with skeletal and dental malocclusion. Observational studies on facial asymmetries among orthodontic patients reported a 12–37 % prevalence of asymmetry among various populations with different anterior and posterior skeletal relationships [13]. Studies on dental malocclusion have found that maximum mandibular asymmetries are observed in patients with anterior crossbite and class II division I malocclusions [6]. Conversely, some authors have shown a high prevalence of mandibular asymmetry among patients with class III dental and skeletal malocclusion compared to class I and II [4,13]. It was postulated that mandibular growth in class III malocclusion is a major risk factor for the development of mandibular asymmetry [14,15]. To eliminate severe mandibular asymmetry, proper treatment should be planned to follow a detailed diagnosis of the growth pattern of mandibular asymmetry.

Evangelista et al. recorded the incidence of mandibular asymmetry in the skeletal sagittal plane and reported that asymmetry in various age groups ranges from 17.50 % to 72.99 %, respectively [1]. Ramirez-Yañez et al. reported that 18.2 % of children in Finland aged 8 to 12 had moderate and severe mandibular asymmetry [16]. In a radiographic study comparing mandibular growth patterns, the authors reported that at the age of 6 years, the left side of the mandible is longer for both genders. However, by the age of 12 years, girls exhibit a longer right side, and for boys, this asymmetry is observed by the age of 16 years [17]. They concluded that there are equal chances for improvement or worsening of the mandible during the developmental phase [17]. Liukkonen et al. concluded that, in general, asymmetry lacks clinical significance and cannot be discerned solely through clinical examination [18]. Moreover, if the asymmetry is significant, it requires orthodontic treatment or surgical improvement. Clinical studies have reported that mandibular asymmetry causes functional difficulties in growing children [11,19]. Even though mandibular asymmetries are commonly reported in growing children, a dimensional change of more than 3 mm between both the sides of the mandible is considered of clinical significance [6,20].

While studies have reported the prevalence of mandibular asymmetry among various populations, there is a scarcity of data regarding its prevalence among healthy children. Till date limited data have been published on mandibular asymmetry among healthy children of Saudi Arabia. It is important to study mandibular growth in this population because it will allow to understand the prevalence and severity of asymmetry within the specific population, which may be influenced by genetic, environmental, and cultural factors particular to Saudi Arabia. Additionally, identifying mandibular asymmetry early in childhood can aid in timely interventions or orthodontic treatments to correct or manage any deviations from normal growth patterns. Finally, such studies contribute to the broader understanding of craniofacial growth and development in diverse populations, which can help maxillofacial surgeons and orthodontics to understand and plan treatment accordingly. Furthermore, no study has evaluated the prevalence of mandibular asymmetry among growing children in Saudi Arabia. Therefore, the present study aims to measure the prevalence of mandibular asymmetry and its fluctuations during the mixed dentition phase in healthy children from the Jazan region of Saudi Arabia. The null hypothesis of this study posits that no statistically significant difference exists when comparing all mandibular linear dimensions in growing children from the Jazan region.

## 2 Method

In this retrospective observational study, the prevalence of mandibular asymmetry was evaluated in the growing children in the Jazan region.

Researchers analyzed dental records from the electronic databases of children who sought treatment at the Pediatric Dental Clinic, College of Dentistry, Jazan, from 2019 to 2022. The inclusion criteria were as follows: 1) healthy children aged 6, 7, and 8 years with mixed dentition, 2) mild class I and class II malocclusion or crowding. Exclusion criteria were: 1) Children having any underlying disease that could have impact mandibular growth, 2) children not opting for orthodontic treatment. Records with unclear OPG images were also excluded, especially in the condylar region. The pre-designed protocol with inclusion-exclusion criteria was prepared and the protocol was submitted to the internal research committee. The internal ethical committee at the College of Dentistry, Jazan University, granted ethical approval for this study (ref no. REC-44/04/354).

Four hundred OPGs were inspected for inclusion by three investigators (M.H·B, T.M.H, and M.S.Z) in consensus. Radiographs of healthy children with no obvious pathologies were selected. Finally, dental radiographs from 390 children (182 boys and 208 girls) were selected. All the radiographs included in this study were captured using a single radiographic machine (Orthopantomogram OP100; Instrumentalism Imaging, Milwaukee, Wisconsin, USA) following standardized protocols.

The OPGs for linear measurements were digitized using a standardized morphometric digitizer (Numonics Cooperation, Washington, USA) and analyzed with Metrix software (System Software, Finland) at a resolution of 300 dpi. The software was pre-calibrated with an 18 mm diameter scale for radiograph scanning. Two sets of mandibular measurements (four linear dimensions) were recorded, along with subjective observations of the mandibular first molar development. The radiographs were traced on 75-μm lacquered polyester acetate tracing paper using a 0.35 mm black lead pencil.

### 2.1 Radiographical measurements

**Fig. 1:**
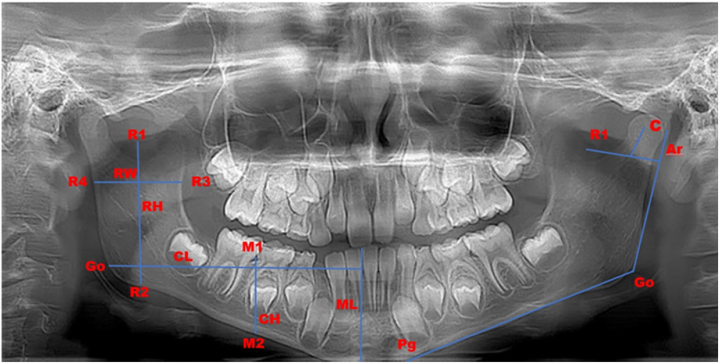
Linear measurements included in this study (R1-R4) 21. C: condylion; Go: gonion; Pg: pogonion; RW: ramus width; RH: ramus height; CL: condylar length; CH: condylar height; ML: mandibular length.

The mentioned linear measurements (Fig. 1) were considered in the radiographical evaluation on both sides.

• *Ramus height (RH):* The measurement along a straight line, starting from the lowest point of the ramus notch (R1) to the lower edge of the mandible (R2), as described by Ricketts [21].
• *Ramus width (RW):* The measurement between the deepest points on the anterior and posterior borders of the ramus (R3 and R4).
• *Condylar height (CH):* The linear measurement from the lowest mesial point on the mandibular first molar at the cementoenamel junction (CEJ) (M1) to the lower border of the mandibular ramus (M2).
• *Condylar length (CL):* The distance between gonion (Go) (the distance from the mandibular plane to the posterior border of the ramus) and mandibular pogonion (pg) (the most anterior point on the mandibular midline) [22].

**Fig. 2:**
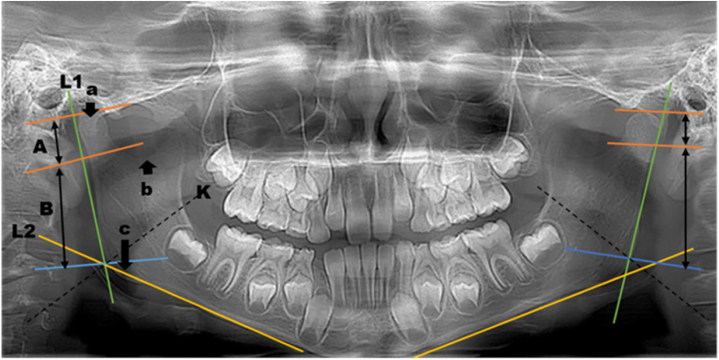
OPG with tangential lines measuring bony points a, b, and c.

Vertically tangential lines (L1 and L2) were drawn to assess mandibular asymmetry (Fig. 2). These lines run parallel to the mandibular corpus. Three bony points (a, b, and c) were selected to measure differences in mandibular growth patterns. Point 'a' represented the prominent condyle point on the panoramic radiograph, while point 'b' indicated the deepest point of the curving incisura. For point 'c,' a line was drawn from a mandibular angle and the perpendicular bisector of the mandibular plane (K). All these points were perpendicular to line L1, and lines A and B were consistently drawn using these bony landmarks.

Two evaluators (MB and AJ) independently performed measurements on the sides of the included radiographs. One evaluator (MB) measured all the parameters on the right side, while another (AJ) on the left side of the radiograph. The reproducibility of measurement was tested by performing similar readings by both the evaluators at the interval of 3 weeks. The intraclass correlation coefficient was measured to evaluate the error between the two measurements.

#### 2.1.1 Calculating distortion factor on the radiograph

Fifteen subjects' orthopantomograms were randomly selected to assess distortion effects. These radiographs were examined by comparing the length of the mandibular first molars on both sides. To do this, an imaginary apical line was drawn through the mesial and distal roots of the first mandibular molar. The length of the molars was measured from the Mesiobuccal cusps to the lowest point at the root apex, perpendicular to the apical line (Fig. 1) (M1-M2). All measurements were taken twice, and a distortion factor was applied to the initial results from both evaluators. Subsequently, these results were used to calculate the asymmetry index (AI), followed by statistical analysis.

### 2.2 Asymmetry index for linear measurement

The AI was utilized to assess the severity of mandibular asymmetry in each subject based on their linear and angular measurements. The index was computed using Saglam's proposed formula:

$$\text{AI}=\frac{Right-Left\;linear\;measurement}{Right+Left\;linear\;measurement}\times 100$$

The calculation was based on percentages: a positive outcome indicated that the right side of the mandible was larger than the left, while a negative outcome indicated the opposite. Mandibular growth was considered symmetric if the percentage equaled 0. Based on the results of previous studies, mandibular asymmetry was categorized as light (3–5%), moderate (5–10 %), and severe (≤10 %) [1,8,17].

### 2.3 Statistical analysis

The final data were analyzed using SPSS version 21.0 (IBM, Chicago, USA). To ensure the reliability of the measurements, intra-examiner correlation was evaluated through the intra-class correlation coefficient (ICC). Descriptive statistics, such as means, percentages, and standard deviations, were computed for the variables of interest. Differences in mandibular growth patterns between the right and left sides were assessed using an independent *t*-test. The Chi-square test was utilized to compare variations in mandibular growth across different age groups. Statistical significance was considered at p ≤ 0.05.

## 3 Results

### 3.1 Evaluator agreements (ICC)

The two evaluators demonstrated a high correlation agreement (r = 0.82) for four linear mandibular measurements. Specifically, the corpus height correlation agreement was (r = 0.93) for the right side and (r = 0.91) for the left side. Ramus height agreement was (r = 0.87) for the right and (r = 0.85) for the left side. The evaluators measured condylar and ramus height (CRH) agreement was (r = 0.86) for the right side and (r = 0.87) for the left side. Finally, condylar length (CL) measurements had a correlation agreement of 0.92 on the right and 0.94 on the left between the evaluators.

### 3.2 Mandibular linear dimensions

The independent *t*-test results indicate statistical significance (p-value≤ 0.05) for both sides of the mandible across two dimensions: CRH (p = 0.03) and mandibular length (ML) (p = 0.04), as shown in Table 1. Table 1 presents the mean and standard deviation for each linear measurement. On the right side of the mandible, the mean condylar height was 21.13 mm, while on the left side, it was 21.23 mm. The mean ramus heights were 33.13 mm for the right side and 32.3 mm for the left side. For other dimensions, the mean CRH was 54.14 mm on the right side and 53.04 mm on the left side, while the ML measured 92.2 mm on the right and 90.52 mm on the left side of the mandible.

**Table 1:** Mean and standard deviation of the mandibular linear measurements.

Variables	Mean	SD	P value
Condylar height (R)^a^	21.13	3.83	0.73
Condylar height (L)^a^	21.23	3.85	
Ramus height (R)	33.13	6.15	0.22
Ramus height (L)	32.6	5.93	
Condylar and ramus height(R)	54.15	7.6	0.03
Condylar and ramus height (L)	53.04	7.3	
Mandible length (R)	92.26	12.18	0.04
Mandible length (L)	90.52	12.23	

Mandibular measurements (in mm) are expressed as Mean ± SD.

a R = right side; L = left side.

**Fig. 3:**
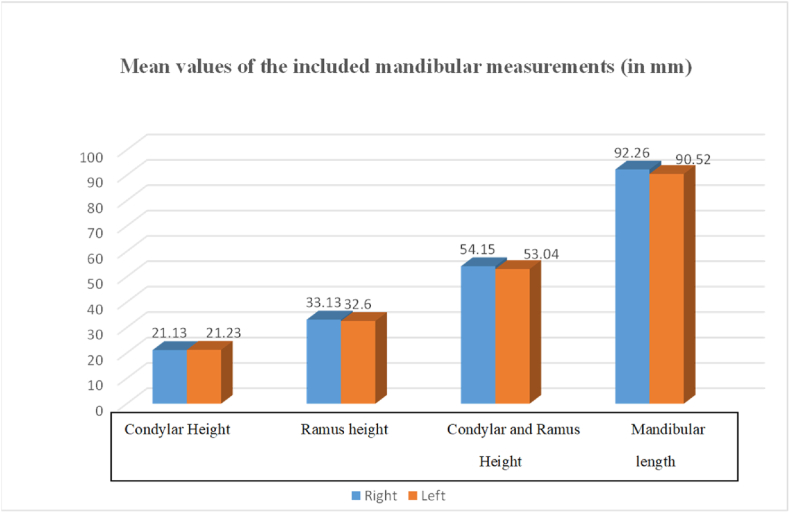
Mean values of the included mandibular measurements.

Table 1 and Fig. 3 depict that Ramus height (33.13 ± 6.5), and condylar and ramus height (54.15 ± 7.6) were greater on the right side, while condylar height (21.23 ± 3.85) and mandibular length (92.26 ± 12.23) were higher on the left side. No statistically significant differences were found in the data concerning age and gender.

### 3.3 Asymmetry analysis

The assessment of mandibular asymmetry using the asymmetry index (AI) revealed no significant asymmetry in most participants when comparing four linear parameters on both sides of the mandible. Out of the 400 subjects, 154 were classified as having severe asymmetry and 108 as having no asymmetry when considering condylar height. In contrast, 134 subjects had no asymmetry, and 128 had severe asymmetry when considering ramus height. Similarly, 202 participants showed no asymmetry while assessing CRH, and 71 had moderate asymmetry. For mandibular length, 236 participants exhibited no asymmetry, and 59 had moderate asymmetry. Light and moderate asymmetry were observed in approximately 14.33 % and 18.25 % of the participants, respectively. This study indicates that more than half of the participants had either no asymmetry (43.7 %) or severe asymmetry (23.71 %) when comparing linear measurements on both sides of the growing mandible. Table 2 presents the number and percentage of participants for each asymmetry category.

**Table 2:** Percentages for the severity of the asymmetry.

Variables	NS (%)^a^	L (%)^a^	M (%)^a^	S (%)^a^
Condylar height	108(27.76)	65(16.71)	62(15.94)	154(39.59)
Ramus height	134(34.45)	35(9)	92(23.65)	128(32.9)
Condylar and ramus height	202(51.93)	66(16.97)	71(18.25)	50(12.85)
Mandible length	236(60.65)	57(14.65)	59(15.17)	37(9.51)
Total	680(43.7)	223(14.33)	284(18.25)	369(23.71)

a NS-no asymmetry; L-light asymmetry; M-moderate asymmetry; S-severe asymmetry.

Table 3 depicts the age-related severity of asymmetry in mandibular development. The result reports that the maximum number of participants showed no asymmetry when compared to four linear dimensions according to age except for condylar height. A high percentage of participants from all the age groups had severe asymmetry when comparing condylar height. The percentage of participants for each linear measurement (CH, CL, CHR, and ML) asymmetry was similar between genders.

**Table 3:** Percentages for the severity of the asymmetry in relation to age.

Variables	Severity of Asymmetry	Age (Years)		
		6	7	8
Condylar height	NS	45(33.3)	28(21.2)	35(28.7)
	L	19(14.1)	14(10.6)	32(26.2)
	M	20(14.8)	18(13.6)	24(19.7)
	S	51(37.8)	72(54.5)	31(25.4)
Ramus height	NS	53(39.3)	37(28)	44(36.1)
	L	9(6.7)	14(10.6)	12(0.8)
	M	36(26.7)	29(22)	27(2.1)
	S	37(27.4)	52(39.4)	39(32)
Condylar and ramus height	NS	77(57)	68(51.5)	57(46.7)
	L	21(15.6)	25(18.9)	20(16.4)
	M	19(14.1)	26(19.7)	26(21.3)
	S	18(13.3)	13(9.8)	19(15.6)
Mandible length	NS	89(65.9)	71(53.8)	76(62.3)
	L	16(11.9)	19(14.4)	22(18)
	M	17(12.6)	25(18.9)	17(13.9)
	S	13(9.6)	17(12.9)	7(5.7)

*NS-no asymmetry; L-light asymmetry; M-moderate asymmetry; S-severe asymmetry.

## 4 Discussion

Mandibular symmetry, or facial symmetry, refers to a balanced equilibrium of size, shape, and pattern in facial structures on opposite sides of the mid-sagittal plane. This study assessed the prevalence of mandibular dimensional asymmetries in children with mixed dentition in the Jazan region. While researchers have documented such asymmetries in young children, some authors argue that these variations are associated with the developmental phase and may not require treatment planning [6,15,23]. The current study partially supports this statement, as most participants showed no asymmetry while measuring all four linear dimensions. However, while comparing corpus height among the age group, the maximum number of participants measured severe asymmetry.

Using orthopantomograms (OPGs) posed a primary concern for researchers due to potential magnification changes when measuring mandibular dimensions. Some studies have suggested that slight variations in head positioning may affect horizontal dimensions, but this effect has not been reported for vertical measurements, making vertical measurements accurate with OPGs [8,17,21]. Consequently, OPGs have been employed in various experimental studies to compare ramus and condylar height in denture wearers, individuals with malocclusions, and those with temporomandibular disorders [3,24]. In an experimental study by Habets et al. OPGs were used as a diagnostic tool to assess temporomandibular disorders in young patients, revealing 6 % mandibular asymmetry on both sides [25]. A few studies support using OPGs as a diagnostic tool for comparing mandibular asymmetries between the right and left sides [17,23,25].

The results of this study support the studies that showed mandibular asymmetries are not associated with age and gender. Nevertheless, oral surgeons and orthodontists are still debating whether these asymmetries shall be considered normal at certain ages [1,23,26,27] and how these asymmetries are associated with developing malocclusions [14,22]. Studies have reported that the asymmetries associated with the growing phase could be due to malocclusion, temporomandibular disorder, and chewing on the preferred side [14,22]. Mandibular deviation during growth appears to be influenced by the modeling and remodeling of the glenoid and condylar fossa, suggesting an adaptive response to functional activity [10,11]. Clinical studies, especially in class III and II malocclusions and cross-bites, indicate that functional shifts can lead to variations in mandibular growth and asymmetries [9,26].

Another noteworthy finding of this study is the significant asymmetry observed in condyle height and ramus height among 7-year-old participants compared to those aged 6 and 8. Similarly, Liukkonen et al.^18^ and Melnik^17^ in their studies observed a significant asymmetry in condylar and ramus growth among the children of age 7 years compared to 16 years. These findings suggest that mandibular asymmetry may fluctuate during the growth period among healthy children. Independent t-tests indicate a high percentage of participants exhibiting mandibular asymmetry, particularly in condyle and ramus height, on the right side compared to the left. The mean values for all four linear measurements (RH, RL, CH, and CL) were consistently higher on the right side of the mandible. These findings align with studies conducted by Kula et al. and Skvarilova, which reported a higher prevalence of asymmetry on the right side among growing children [9,28]. However, it is essential to note that these results contradict the study by Ramirez-Yañez et al., where left-side asymmetry was more prominent in young children [16]. This variation in findings may be attributed to differences in the populations studied. Nonetheless, based on the current study and previously published research, it can be generalized that mandibular asymmetry tends to be more prevalent on the right side in young children.

The current study also recorded statistically significant mandibular asymmetry across two dimensions: CRH (p = 0.03) and ML (p = 0.04). A closer observation showed that the condylar and ramus height and mandibular length measure differently in the growing stages. Although there is a considerable difference in condylar and ramus heights and mandibular length between the right and left, the clinical relevance of this remains uncertain. Mandibular asymmetry is not a clinical problem until it is in severe form, affecting the esthetics of young children. Interestingly, none of the subjects in this study returned to the dental clinic due to facial asymmetry problems. The decision to initiate treatment of patients with severe asymmetry should be carefully considered. This decision should always be aligned with the findings of Melnik [17], suggesting that mandibular asymmetry can either diminish or become more prominent during the growth phase in healthy children. This fluctuation during growth may indicate an imbalance in the functional forces affecting the joints and mandibular gonial regions, leading to uneven growth in condyle and ramus heights on the right and left sides [10,24]. No definitive calculation indicates the need for treatment. Instead, the decision should be based on the likelihood that facial asymmetry may result in functional or aesthetic issues for the individual. Furthermore, in cases of only mild asymmetry observed on orthopantomograms, when the subject exhibits other indications of conditions (such as TMDs, cross-bites, malocclusions, and hemifacial microsomia), it is essential to utilize additional diagnostic tools, such as magnetic resonance imaging and/or computed tomography scans, for a comprehensive diagnosis and follow-up [29].

Robust statistical testing and significant sample size of children in Jazan was the strength of the current study. The main purpose of this study was to provide dental professionals with a better understanding of the factors associated with the mandibular asymmetries. The result of this study indicates that analysis of mandibular asymmetry is necessary for orthognathic surgery and orthodontic patients since its prevalence is high among growing children. If left untreated mandibular asymmetry might lead to a compromised outcome. Evaluating the linear dimension was the main weakness of this study, which could have influenced the findings.

## 5 Limitations and further recommendations

To author knowledge, this is the first study to measure mandibular asymmetry in growing children which is the strength of this study. However, this study has several limitations. Firstly, its findings cannot be generalized since the participant pool was limited to those visiting the dental school. Therefore, studies on the prevalence of mandibular asymmetries among young children should be planned further to acknowledge the impact of these asymmetries on facial growth. Secondly, the study focused solely on linear dimensions and did not include angular measurements, which could have influenced the results. Thirdly, the diagnostic aid used was OPGs, and it is important to note that diagnostic errors and variations in radiographic measurements can occur. Lastly, the data were retrospective, introducing the possibility of distortion. To build on these findings, future studies should involve larger and more diverse samples of young children from various regions in Saudi Arabia. Additionally, using 3D scanners could enhance the accuracy of measurements for the mandible's linear and angular dimensions.

## 6 Conclusion

Within the limitations of this study, it could be concluded that there is a statistical significance in the mean difference of the condylar and ramus height among all the age groups included in the Jazan region. Moreover, dimensional asymmetry was recorded among the condylar and ramus heights of 7 years, indicating variation in growth patterns among age groups. However, no significant difference was recorded among the genders. It is important to note that the subjects in this study did not have any pathologies or disorders associated with variation in mandibular growth patterns. Hence, it could be hypothesized that even though significance was recorded in a few measurements, it does not influence the well-being of the patients. Maxillofacial surgeons and orthodontists should be cautious while planning the treatment for mandibular asymmetry as this condition as mild to moderate asymmetry diminishes or appears after growing age. Therefore, further studies are required to establish a better association between mandibular asymmetries among growing children and oral functions.

## Statement of clinical relevance

Asymmetries in the mandible can result from the adaptive response of the mandible during growth phase. Understanding radiographical characteristics of mandibular development can help to improve the diagnostic accuracy for surgical corrections and orthodontic requirements of the children.

## Ethical consideration

The internal ethical committee at the College of Dentistry, Jazan University, granted ethical approval for this study (ref no. REC-44/04/354). Informed consent was not required for this study as it was a retrospective study on orthopantomogram of patients.

## Data availability

Data utilized in this study is publicly available and can be assessed from Dr. Mohammed Mousa H. Bakri, Email: mmb644@nyu.edu.

## Source of funding

None.

## CRediT authorship contribution statement

**Mohammed Mousa H. Bakri:** Writing – review & editing, Writing – original draft, Methodology, Investigation, Funding acquisition, Formal analysis, Conceptualization. **Satish Vishvnathaiah:** Visualization, Conceptualization. **Haifa Fathuldeen Bakmani:** Supervision, Resources, Methodology. **Abdullah Jaber Hakami:** Writing – review & editing, Writing – original draft, Project administration, Conceptualization. **Meshal Saleh Zaidan:** validation. **Mohammed Abdullah Dighriri:** Validation. **Yaser Ali Jad:** Software, Investigation. **Thamer Mohammad Hakami:** Software. **Hamed Mousa H. Bakri:** Supervision, Funding acquisition, Formal analysis, Data curation, Conceptualization.

## Declaration of competing interest

The authors declare that they have no known competing financial interests or personal relationships that could have appeared to influence the work reported in this paper.

## References

[1] Evangelista K. (2022). Prevalence of mandibular asymmetry in different skeletal sagittal patterns. Angle Orthod..

[2] Kilic N., Kiki A., Oktay H. (2008). Condylar asymmetry in unilateral posterior crossbite patients. Am. J. Orthod. Dentofacial Orthop..

[3] Sobieska E., Walerzak M., Molińska M. (2020). Forum Ortodontyczne/Orthodontic Forum.

[4] Jhamb T. (2023). Three-dimensional morphometric analysis of anterior cranial base in growing patients. Am. J. Orthod. Dentofacial Orthop..

[5] Lee S.K. (2001).

[6] Azevedo A.R. (2006). Evaluation of asymmetries between subjects with Class II subdivision and apparent facial asymmetry and those with normal occlusion. Am. J. Orthod. Dentofacial Orthop..

[7] Hesse K.L. (1997). Changes in condylar postition and occlusion associated with maxillary expansion for correction of functional unilateral posterior crossbite. Am. J. Orthod. Dentofacial Orthop..

[8] Kheir N.A., Kau C.H. (2014). Measuring mandibular asymmetry in Class I normal subjects using 3D novel coordinate system. Ann Maxillofac Surg.

[9] Kula K., Esmailnejad A., Hass A. (1998). Dental arch asymmetry in children with large overjets. Angle Orthod..

[10] Nicot R. (2020). Condyle modeling stability, craniofacial asymmetry and ACTN3 genotypes: contribution to TMD prevalence in a cohort of dentofacial deformities. PLoS One.

[11] Obwegeser H.L., Luder H.-U. (2000).

[12] Van Elslande D.C. (2008). Mandibular asymmetry diagnosis with panoramic imaging. Am. J. Orthod. Dentofacial Orthop..

[13] Vieira A.R. (2019). Orthodontics and genetics. Dental Press Journal of Orthodontics.

[14] Haraguchi S., Iguchi Y., Takada K. (2008). Asymmetry of the face in orthodontic patients. Angle Orthod..

[15] Forsberg C.T., Burstone C.J., Hanley K.J. (1984). Diagnosis and treatment planning of skeletal asymmetry with the submental-vertical radiograph. Am. J. Orthod..

[16] Ramirez-Yañez G.O. (2011). Prevalence of mandibular asymmetries in growing patients. Eur. J. Orthod..

[17] Melnik A.K. (1992). A cephalometric study of mandibular asymmetry in a longitudinally followed sample of growing children. Am. J. Orthod. Dentofacial Orthop..

[18] Liukkonen M., Sillanmäki L., Peltomäki T. (2005). Mandibular asymmetry in healthy children. Acta Odontol. Scand..

[19] Babczyńska A., Kawala B., Sarul M. (2022). Genetic factors that affect asymmetric mandibular growth—a systematic review. Symmetry.

[20] Frost H.M. (2004). A 2003 update of bone physiology and Wolff's Law for clinicians. Angle Orthod..

[21] Ricketts R.M. (1961). Cephalometric analysis and synthesis. Angle Orthod..

[22] Joondepha D.R. (2000). Mysteries of asymmetries. Am. J. Orthod. Dentofacial Orthop..

[23] Edler R., Wertheim D., Greenhill D. (2003). Comparison of radiographic and photographic measurement of mandibular asymmetry. Am. J. Orthod. Dentofacial Orthop..

[24] Uysal T. (2009). Condylar and ramal vertical asymmetry in unilateral and bilateral posterior crossbite patients and a normal occlusion sample. Am. J. Orthod. Dentofacial Orthop..

[25] Habets L.L. (1987). The orthopantomogram, an aid in diagnosis of temporomandibular joint problems. I. The factor of vertical magnification. J. Oral Rehabil..

[26] Duthie J. (2007). A longitudinal study of normal asymmetric mandibular growth and its relationship to skeletal maturation. Am. J. Orthod. Dentofacial Orthop..

[27] Ferrario V.F. (2001). The effect of sex and age on facial asymmetry in healthy subjects: a cross-sectional study from adolescence to mid-adulthood. J. Oral Maxillofac. Surg..

[28] Skvarilova B. (1993). Facial asymmetry: type, extent and range of normal values. Acta Chir. Plast..

[29] Kambylafkas P. (2006). Validity of panoramic radiographs for measuring mandibular asymmetry. Angle Orthod..

